# Late Presentation of a 9 mm Bullet in the Ureteropelvic Junction Causing Acute Renal Failure in a Solitary Functioning Left Kidney

**DOI:** 10.1089/cren.2018.0068

**Published:** 2018-10-01

**Authors:** Hasan Jhaveri, Michael D'Angelo

**Affiliations:** ^1^ University of Florida College of Medicine, Gainesville, Florida.; ^2^ Heart of Florida Regional Medical Center, Davenport, Florida.

**Keywords:** percutaneous nephrolithotomy, urinary obstruction, abdominal gunshot injury, hydronephrosis, acute renal failure, renal calculus

## Abstract

***Background:*** Urinary obstruction as a result of a late complication of a gunshot wound is rarely reported. Bullet shell fragments may migrate from their initial location into an area causing obstruction. In this study, we present a case of a left renal calculus surrounding a 9 mm gun shell in a patient with a solitary functioning left kidney.

***Case Presentation:*** A 53-year-old man presented with left hydronephrosis found on an urgent CT scan following complaints of left flank pain and signs of acute renal failure 10 years after suffering a gunshot wound to the abdomen. Urgent cystoscopy and bilateral retrograde pyelograms revealed a left ureteropelvic junction calculus surrounding a 9 mm bullet fragment that was ultimately removed percutaneously.

***Conclusion:*** Abdominal gunshot shell fragments may migrate over time causing urinary obstruction. In this case the past medical history of the previous gunshot wounds provided insight into the etiology of the patient's actual clinical presentation. This led to the best endoscopic approach for the effective treatment of this unique case.

## Introduction and Background

Urinary obstruction due to late complications of abdominal gunshot wounds may be difficult to diagnose. Ureteral obstruction caused by gun shells or bullets has seldom been reported. In those rare cases, the foreign bodies have passed spontaneously without the need for further intervention.^1^ Ureteral obstruction secondary to blood clots after gunshot injury has also been reported.^2^ Hydronephrosis and acute renal failure as a result of late complications of traveling bullet fragments into a solitary functioning kidney have never been reported. We present a case of a left renal calculus surrounding a 9 mm gun shell obstructing the ureteropelvic junction (UPJ) from a gunshot injury from 10 years prior in a patient with a solitary functioning left kidney.

## Presentation of Case

**FIG. 1.**
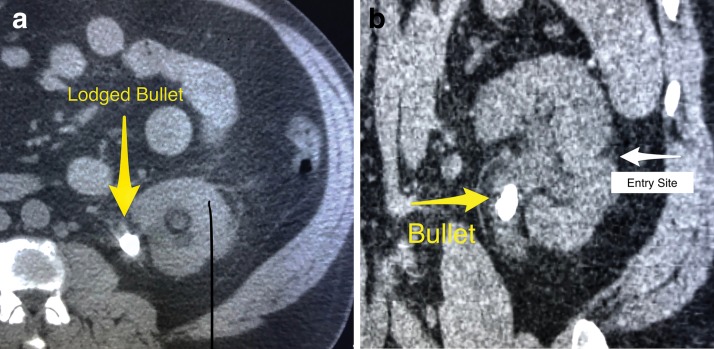
CT scan image indicates a bullet fragment in the right UPJ **(a,** transverse view; **b,** sagittal view**)**. UPJ = ureteropelvic junction.

A 53-year-old male first appeared in the emergency department complaining of a 3-day history of left flank pain and acute renal failure with no other associated symptoms. His past medical history included three gunshot wounds on separate occasions, the last one over 10 years ago with a bullet lodged in the left kidney. His past surgical history included a right pulmonary lobectomy and multiple bowel surgeries with mesh placement from a prior gunshot wound that resulted in injury of the right kidney. After partial right nephrectomy, the patient showed renal insufficiency with a creatinine of 2.6 mg/dL. After the latest gunshot wound, no attempt at removal of the left sided bullet was performed due to risk of injury to the solitary functioning left kidney. Physical examination findings were unremarkable. However, acute renal failure was diagnosed with a blood urea nitrogen of 39 mg/dL and a creatinine of 5.0 mg/dL. The CT scan revealed hydronephrosis of the left kidney and a 15 mm calcification in the left UPJ and an atrophic right kidney (Fig. 1a, b). The patient then underwent urgent cystoscopy with bilateral retrograde pyelograms showing a filling defect consistent of a calculus in the left UPJ causing hydronephrosis. The patient underwent left ureteral stent placement with plans for either ureteroscopic laser lithotripsy or extracorporeal shockwave lithotripsy (SWL).

**FIG. 2.**
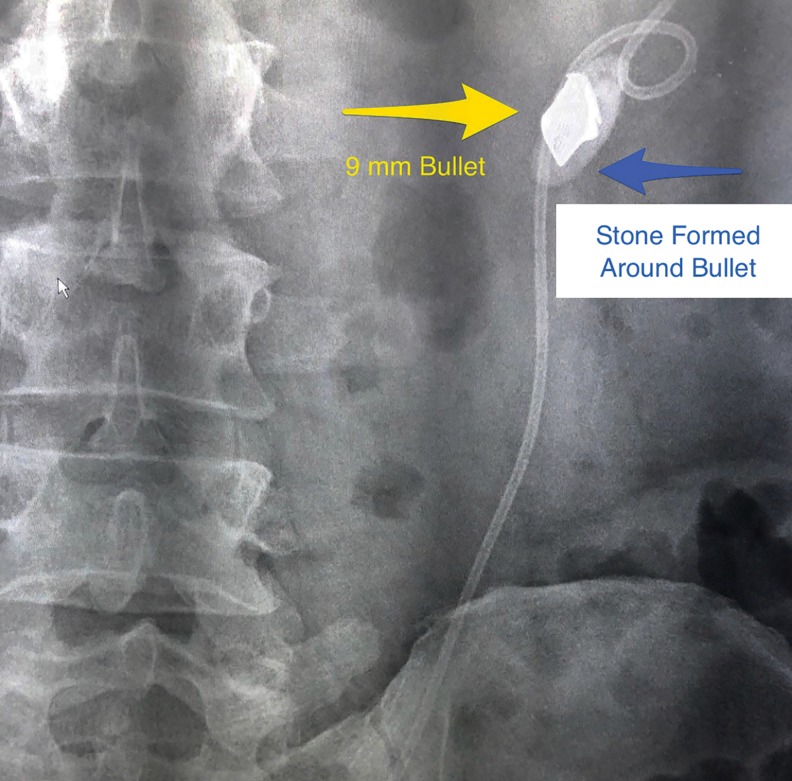
Fluoroscopic image of bullet fragment noted in the right UPJ with Double-J ureteral stent noted in place.

With left renal decompression, the patient's renal failure improved with creatinine dropping to a low of 1.68 mg/dL, and the patient symptoms of left flank pain improved. Upon planning the elective procedure, review of the films with radiologist in context of the patient's prior history of a left gunshot wound indicated the high possibility that the calcification in the left UPJ was actually the bullet possibly surrounded by stone formation (Fig. 2). Therefore, ureteroscopic laser lithotripsy by a retrograde approach and SWL were contraindicated.

**FIG. 3.**
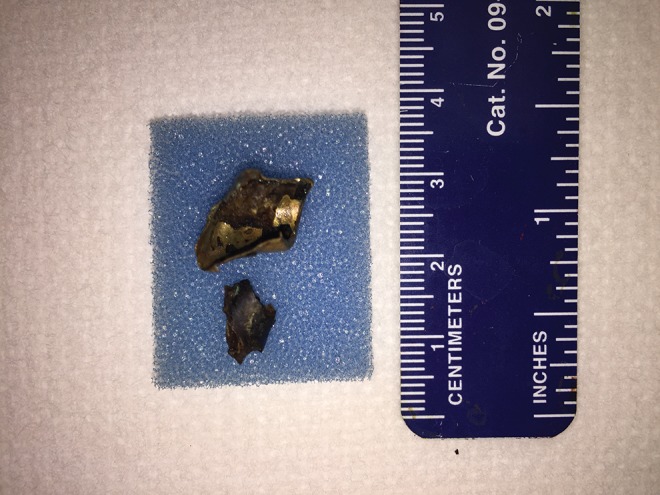
Bullet fragment specimens postextraction.

The patient then underwent left percutaneous ultrasonic lithotripsy of the calcified stone formation over the bullet and endoscopic removal of the bullet shell. The ultrasonic probe was used to chip off stone fragment surrounding the gun shell. Several large stone chunks were removed. Using a 100 W holmium laser, the final remaining stone fragments were removed from around the bullet. Due to the inability of the laser to fragment the metal, the gun shell shard was grasped, pulled into the sheath, and the sheath and fragment were removed together (Fig. 3).

The patient had an unremarkable recovery and was discharged 2 days after the procedure.

## Discussion and Literature Review

Late complications of abdominal gunshot wounds are difficult to predict. It is likely since the time of the left-sided gunshot wound the bullet migrated from the collecting system of the kidney to the UPJ causing the patient's left flank pain and acute renal failure. The presentation of left renal colic initially appeared to be caused by a left renal pelvic stone. Upon consultation with the radiologist in context with his prior history of a left-sided gunshot wound, we determined that the obstruction of the left kidney was caused by a bullet that migrated into the UPJ with subsequent calcification.

Laparotomy or laprascopic/robotic approaches for removal of complex renal calcifications caused by foreign bodies may prove beneficial for patients with similar diagnoses.^3^ However, due to our patient's unique and extensive previous surgical history, including bowel repair and mesh placement, these approaches would be less favorable options. Retrograde ureteroscopic approaches are considered optimal treatment options for lithiasis involving the lower third of the ureter^3^ and currently are in use for many upper ureter and renal stones. However, due to the inability to fragment the 9 mm bullet shell, this retrograde approach was ruled out. We conducted a percutaneous laser lithotripsy in treatment of the outer calcified component. We then had to remove the bullet fragment in its entirety through the larger access of the percutaneous tract. Percutaneous approaches in treating abdominal trauma have been implicated previously in the literature.^4^

One concern for the percutaneous approach was in removal of the complete 9 mm bullet through a 10 mm Amplatz sheath. Neither the ultrasound nor the laser would be able to fragment the metal bullet. Therefore, the entire bullet was wedged into the sheath, and the sheath with the bullet could then be removed simultaneously through the percutaneous tract.

## Conclusion

Abdominal gunshot shell fragments may migrate over time causing urinary obstruction. We have presented a novel case of a left renal calculus surrounding a 9 mm bullet in a patient with a solitary functioning left kidney. In this case the past medical history of the previous gunshot wounds provided insight into the etiology of the patient's actual clinical presentation. This led to best endoscopic approach for the effective treatment of this unique case.

## Abbreviations Used

CT: computed tomography
SWL: extracorporeal shockwave lithotripsy
UPJ: ureteropelvic junction

## References

[1] Macwilliam JL, Mistry R, Floyd MS Jr. Delayed ureteral obstruction following gunshot pellet migration. Urol J 2013;10:847 23801465

[2] Schreiber MA, Coburn M Complete upper and lower urinary tract obstruction caused by penetrating pellet injury of the kidney. J Trauma 2001;50:1144–1146 11426132 10.1097/00005373-200106000-00027

[3] Fernandes ET, Wrenn E Jr, Jerkins G, Noe HN Late urologic complication of an abdominal gunshot wound. J Pediatr Surg 1990;25:1283–1284 2286909 10.1016/0022-3468(90)90533-f

[4] Taqi KM, Nassr MM, Al Jufaili JS, Abu-Qasida AI, Mathew J, Al-Qadhi H Delayed diagnosis of ureteral injury following penetrating abdominal trauma: a case report and review of the literature. Am J Case Rep 2017;18:1377–1381 29273706 10.12659/AJCR.905702 PMC5747955

